# Supplementary material for: Lactobacillus delbrueckii Ameliorated Blood Lipids via Intestinal Microbiota Modulation and Fecal Bile Acid Excretion in a Ningxiang Pig Model
Source: Animals (Basel). 2024 Jun 17;14(12):1801. doi: 10.3390/ani14121801 (PMC11201289; doi:10.3390/ani14121801)
Supplement: Supplementary file 1 [file animals-14-01801-s001.zip › animals-3007678-supplementary.pdf]

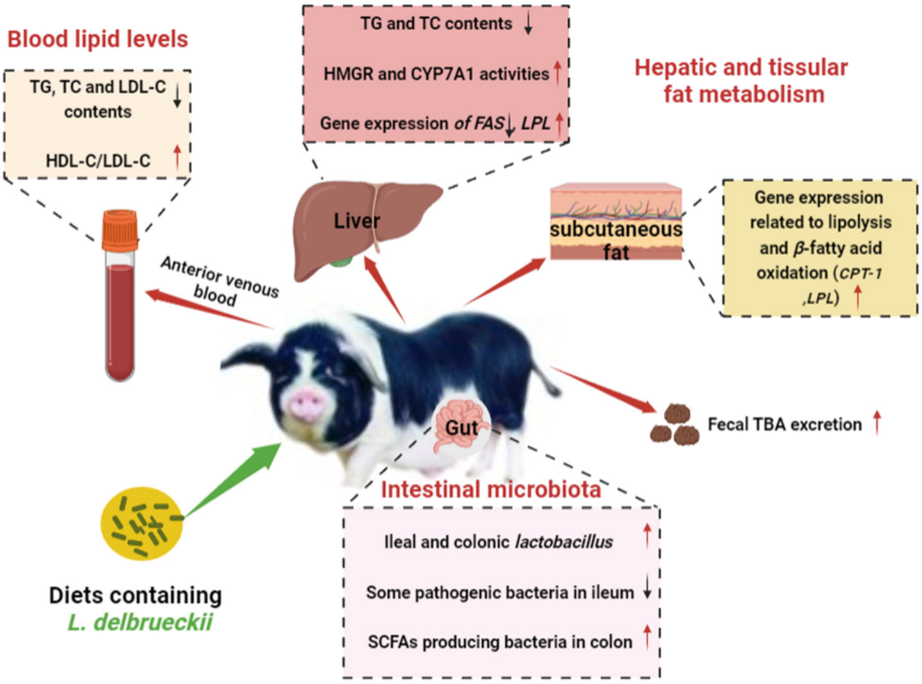

Figure S1. Underlying mechanism by which *L. delbrueckii* lowers blood lipid levels in Ningxiang pigs. *L. delbrueckii* regulates hepatic and tissular fat metabolism via gut microbiota modulation and fecal TBA excretion to reduce blood lipid levels in Ningxiang pigs. The up red arrow means an increase, while the down black arrow indicates a decrease.

Table S1. Microbiota Composition at Phylum and Genus Level in Ileal Digesta

Items	CON	LD	*P* value
Phylum level			
Firmicutes	0.8113±0.1783	0.7452±0.2119	0.706
Proteobacteria	0.1839±0.1792	0.2514±0.2108	0.714
Actinobacteria	0.0042±0.0033	0.0020±0.0001	0.426
Bacteroidetes	0.0002±0.0002	0	0.051
Tenericutes	0.0001±0.0001	0.0002±0.0003	0.163
Candidatus Saccharibacteria	0.0002±0.0004	0.0011±0.0014	0.129
Cyanobacteria/Chloroplast	0.0001±0.0001	0	0.280
Spirochaetes	0	0	0.024
WPS-2	0	0	
Fibrobacteres	0	0	
Genus level			
Clostridium XI	0.2532±0.1523	0.2682±0.1417	0.846
Escherichia/Shigella	0.1460±0.1267	0.2439±0.2094	0.320
Clostridium sensu stricto	0.4024±0.2549	0.3272±0.1894	0.483
Pasteurella	0.0143±0.0269	0.0020±0.0021	0.033
Actinobacillus	0.0134±0.0225	0.0055±0.0037	0.050
Haemophilus	0.0090±0.0157	0.0010±0.0018	0.041
Streptococcus	0.0164±0.0073	0.0408±0.0405	0.001

Turicibacter	0.0935±0.0583	0.0702±0.0626	0.600
Veillonella	0.0029±0.0028	0.0012±0.0011	0.065
Lactobacillus	0.0026±0.0014	0.0114±0.0170	0.038
unclassified	0.0140±0.0116	0.0172±0.0119	0.787
Campylobacter	0.0002±0.0004	0	0.032
Klebsiella	0.0007±0.0005	0.0008±0.0008	0.094
Coprococcus	0.0115±0.0127	0.0045±0.0039	0.166
Ruminococcus	0.0005±0.0003	0.0001±0.0001	0.298
Cellulosilyticum	0.0158±0.0143	0.0044±0.0025	0.004
Gemella	0.0005±0.0002	0.0006±0.0007	0.164
Helicobacter	0.0001±0.0001	0	0.003
Corynebacterium	0.0024±0.0018	0.0009±0.0005	0.081
Sharpea	0.0001±0.0001	0.0001±0.0001	0.930
Advenella	0.0001±0.0001	0.0001±0	0.054
Aeromonas	0	0	0.024
Prevotella	0.0001±0.0001	0	0.000
Actinomyces	0.0002±0.0002	0.0003±0.0004	0.347
Barnesiella	0	0	0.002

Table S2. **Microbiota Composition at Phylum and Genus Level in Colonic Digesta**

Items	CON	LD	*P* value
Phylum level			
Firmicutes	0.7634±0.0654	0.6853±0.0776	0.743
Bacteroidetes	0.1667±0.0677	0.2222±0.0591	0.936
Spirochaetes	0.0261±0.0166	0.0473±0.0207	0.512
Actinobacteria	0.0196±0.0053	0.0180±0.0066	0.348
Proteobacteria	0.0119±0.0119	0.0163±0.0080	0.128
Tenericutes	0.0055±0.0026	0.0038±0.0012	0.010
WPS-2	0.0010±0.0010	0.0003±0.0003	0.001
Fibrobacteres	0.0006±0.0003	0.0008±0.0005	0.221
Candidatus Saccharibacteria	0.0019±0.0021	0.0006±0.0005	0.069
Cyanobacteria	0.0035±0.0029	0.0055±0.0038	0.306
Genus level	0.7634±0.0654	0.6853±0.0776	0.743
unclassified	0.6288±0.0647	0.5431±0.0379	0.395
Blautia	0.0608±0.0410	0.0884±0.0354	0.771
Treponema	0.0213±0.1270	0.0173±0.0134	0.952
Lactobacillus	0.0216±0.0133	0.0311±0.0191	0.065
Ruminococcus	0.0123±0.0164	0.0241±0.0127	0.658
Coprococcus	0.0374±0.0080	0.0266±0.0083	0.740
Oscillibacter	0.0363±0.0130	0.0317±0.0078	0.004

Clostridium XlVa	0.0264±0.0106	0.0429±0.0100	0.964
Prevotella	0.0216±0.0045	0.0186±0.0043	0.736
Alloprevotella	0.0224±0.0072	0.0286±0.0076	0.875
Phascolarctobacterium	0.0205±0.0054	0.0194±0.0078	0.156
Lachnospiracea_incertae_sedis	0.0273±0.0149	0.0279±0.0149	0.824
Eubacterium	0.0042±0.0036	0.0152±0.0154	0.096
Erysipelotrichaceae_incertae_sedis	0.0050±0.0031	0.0042±0.0033	0.620
Anaerovibrio	0.0098±0.0033	0.0084±0.0011	0.001
Clostridium XI	0.0071±0.0034	0.0082±0.0041	0.672
Roseburia	0.0057±0.0044	0.0039±0.0023	0.194
Clostridium IV	0.0076±0.0032	0.0303±0.0416	0.056
Butyricicoccus	0.0070±0.0004	0.0088±0.0076	0.032
Streptococcus	0.0044±0.0025	0.0095±0.0109	0.073
Dorea	0.0030±0.0023	0.0062±0.0085	0.087
Ruminococcus2	0.0069±0.0030	0.0044±0.0006	0.065
Oscillospira	0.0028±0.0022	0.0014±0.0024	0.732
